# The role of microRNAs during the genesis of medulloblastomas induced by the hedgehog pathway

**DOI:** 10.1016/S1674-8301(11)60005-5

**Published:** 2011-01

**Authors:** Xiaoju Luo, Jun Liu, Steven Y Cheng

**Affiliations:** Department of Developmental Genetics and Center for Regenerative Medicine, Nanjing Medical University, Nanjing, Jiangsu 210029, China

**Keywords:** Hedgehog signaling pathway, microRNAs, miR-154 cluster, medulloblastomas

## Abstract

Constitutive hedgehog (Hh) signaling is associated with the genesis of medulloblastomas (MB). The objective of this study is to identify special microRNAs (miRNAs) regulated by the Hh pathway, and to clarify the role of miRNAs during the genesis of MB induced by sustained Hh activation. In the primary screening, we used stem-loop RT-PCR to test the expression of 90 different miRNAs in the wildtype (WT) and Ptc-/- MEF cell lines. In the secondary screening, the miRNAs screened from the first screening were validated in the Sufu-/- MEF cell lines. We then verified the expression of miRNAs both in the normal cerebellar tissues and the MB induced by activated Hh pathway, and examined the expression of the other 21 miRNA members of the *miR*-*154* cluster in the MB and normal cerebellum. In the first screening, 13 miRNAs showed significant differential expression in WT and Ptc-/- MEF cell lines, while 10 of them had significant difference in the Sufu-/- MEF cell line. Compared to the normal mouse cerebellum, only 2 miRNAs in 15 miRNAs were differentially expressed between the MB and normal cerebellar tissues. Among 21 members of the *miR*-*154* cluster, 6 miRNAs were downregulated in the MB. Our study demonstrated that *miR*-*154* may be regulated by the Hh pathway, and the activation of the Hh pathway led to the downregulation of the *miR*-*154* cluster, resulting in the genesis of MB.

## INTRODUCTION

Hedgehog (Hh) is one of the primary morphogenic signals that specify the patterns of cell growth and differentiation during vertebrate development. Sustained Hh signaling pathway activity can lead to congenital anomalies and development of various tumors[1]. Medulloblastomas (MB), which are aggressive childhood brain tumors of cerebellar origin, are associated with inappropriate Hh pathway activity[2]–[5]. MB is a “stem cell” tumor caused by the abnormal proliferation of granular cells in the internal granule layer (IGL) of the cerebellum, which come from the differentiation of granular cell precursors (GCPs) in the external germinal layer (EGL)[6]–[7]. In general, the GCPs stop dividing and exit the cell cycle to keep the number of granular cells at a stable level one month after birth. When the Hh pathway is abnormally activated, GCP cells cannot exit the cell cycle; however, they continue to proliferate and form a large number of granule cells accumulating in the EGL layer, and finally develop MB. The mechanism of this pathological process remains elusive[8]–[9].

MicroRNAs (miRNAs) are a group of small noncoding RNAs that are about 22-nucleotide in length and can silence gene expression by mRNA degradation or translation inhibition through binding to the 3′ untranslated region (3′UTR) of target mRNAs[10]–[12]. The relationship between the Hh signaling pathway and miRNAs has been reported. Flynt *et al*.[13] discovered that *miR*-*214* targeted a Hh pathway suppressor gene *Sufu* to regulate somite development in zebrafish. Lee *et al*.[14] reported that *miRNA*-*378* promoted cell survival, tumor growth and angiogenesis by targeting *SuFu*. Paul *et al*.[15] showed that the *miRNA17*-*92* cluster collaborated with the Hh pathway in medulloblastoma. In the present study, we chose 90 miRNA candidates to screen out the miRNAs probably regulated by the Hh signaling pathway and to further clarify the role of miRNAs in the genesis of MB induced by abnormal Hh signaling pathway activity.

## MATERIALS AND METHODS

### Cell culture

Wildtype (WT) mouse embryonic fibroblast (MEF), Ptc-/- MEF and Sufu-/- MEF cells were maintained in DMEM containing 100 U/mL penicillin-streptomycin supplemented with 10% fetal bovine serum (FBS, Gibco, USA) at 37°C in 100-mm cell culture dishes (Corning, USA) in a humidified atmosphere of 5% CO_2_. When cells reached confluence, they were washed with 0.01 mol/L PBS, digested with 0.25% trypsin-EDTA for 2 min, centrifuged at 1,500 *g* for 3 min, and then resuspended in culture medium.

### Animals

*Ptc* and *p53* gene knockout C57/B6 mice (gifts from the Jackson laboratory) were housed in a temperature- and humidity-controlled room and maintained on a 12-h light-dark cycle with access to food and water *ad libitum*. These two kinds of knockout mice (*Ptc* and *p53* gene knockout mice) were mated to form hybrids, respectively.

### Design of RT-PCR primers

Mature miRNAs were amplified by the method of stem-loop RT-PCR, which was firstly invented and reported by Chen *et al*.[16] Through adding an additional stem-loop structure at the 5′' end of the reverse transcription primers, it can increase the length of cDNA and steric hindrance to increase the specificity of PCR amplification of short RNA fragments. The PCR primers of *Gli1* and *GAPDH* were designed using the Primer 5.0 software. The primer sequences were: Gli1: 5′-TCCAGCTTGGATGAAGGACCTTGT-3′ (sense) and 5′-GCATATCTGGCACGGAGCATGTA-3′ (antisense); GAPDH: 5′-ACCCAGTCCTCACCTTCCAC-3′ (sense) and 5′- GGCCTCCTCTTTCTCCCAC-3′ (antisense).

### RNA isolation and RT-PCR

Total RNAs were extracted from MEF cells and mouse normal and tumor tissues with RNAiso Plus reagent (Takara, Japan) according to the manufacturer's instructions. A 0.5 µg aliquot of total RNAs was reverse transcribed into cDNA using the stem-loop reverse transcript primers mentioned before. Then, mature miRNAs were amplified by PCR using the appropriate primers. The PCR was carried out in a total volume of 20 µL containing 20 mmol/L Tris-HCl, 50 mmol/L KCl, 1.5 mmol/L MgCl_2_, 0.2 mmol/L dNTPs, 0.6 mmol/L of forward and reverse primers, and 2.5 U of *Taq* DNA polymerase. The housekeeping gene-*GAPDH* was used as an internal control. *MiR*-*10a* and *miR*-*125b* were the internal controls of miRNAs. PCR was done in PCR tube, and the amplification cycles of Gli1 and GAPDH were 35 and 26, respectively. Denaturing, annealing, and extension reactions were performed at 94°C for 30 s, 55°C for 30 s, and 72°C for 45 s, respectively. Amplification condition of miRNAs was 95°C for 5 min followed by 40 cycles of 95°C for 15 s and 60°C for 1 min.

### Mouse breeding and extraction of medulloblastomas

Mice homozygous for *Ptc* mutation died before E9.5 because of closed neural tube defects. We got Ptc+/- mice through mating male Ptc+/- and female Ptc+/- mice. Ptc+/- mice were also crossed with p53-/- mice to get p53+/-; Ptc+/- or p53-/-; Ptc+/- mice. The status of mice was carefully observed during the experiment process. As soon as the anomalous cerebellar symptoms such as skull enlargement, slow response or ataxia appeared, the mice were sacrified and the tumor tissues were removed for total RNAs isolation. Compared to the normal cerebellums, the tumor tissues showed increased volume and flattened superficial sulcus.

The animal protocal was approved by the local institation review board and animal study was carried out in accordance with the established guidelines for experimental animal use.

### Statistical analysis

Values were shown as mean±SD. The primary screening was performed by one-way ANOVA and Bonferroni adjustment. *P* value < 0.017 was considered statistically significant. Student's *t*-test was used to test the expression of the *miR*-*154* cluster. *P* value < 0.05 was considered statistically significant. All the statistical analyses were performed with SPSS11.0 (SPSS InC., USA).

## RESULTS

### Screening of miRNAs regulated by the Hh signaling pathway in WT MEF, Ptc-/- MEF, and Sufu-/- MEF cell lines

**Fig. 1:**
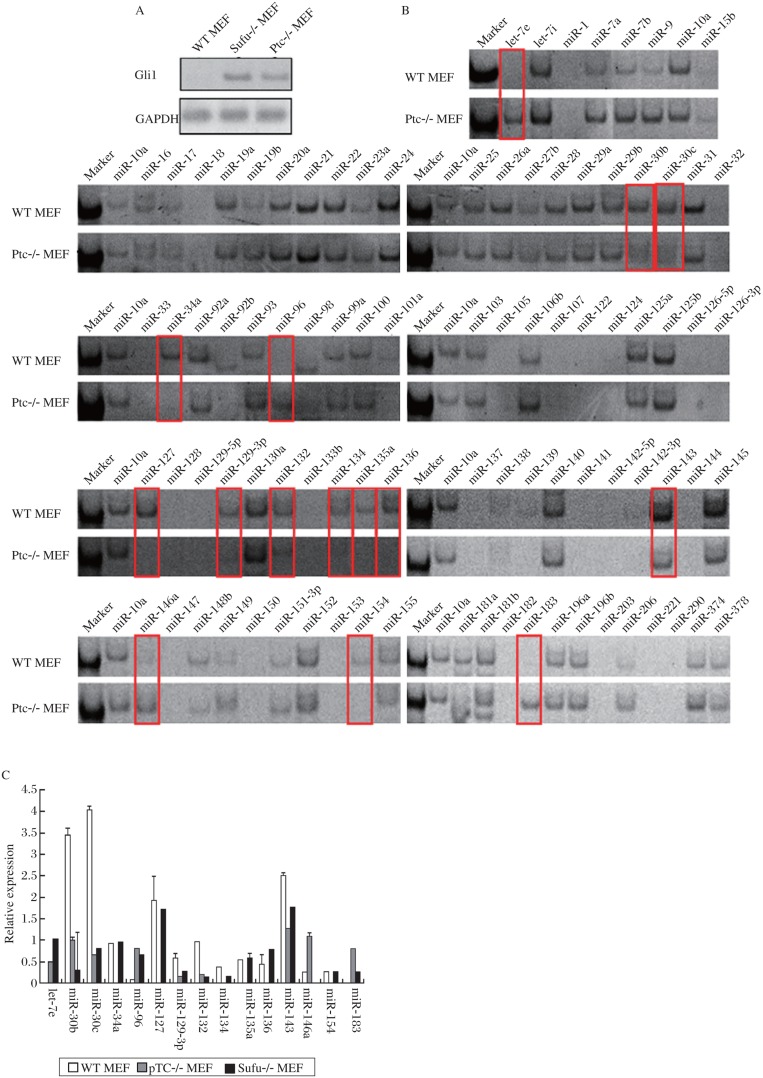
Expression profile of 90 miRNAs in the MEF cell lines. A: The hedgehog signaling pathway was activated in both Ptc-/- and Sufu-/- MEF cell lines. Total RNAs were isolated from the MEF cell lines and the mRNA levels of Gli1 were detected by RT-PCR. GAPDH was used as an internal control. B: Expression of 90 miRNAs was tested in WT MEF and Ptc-/- MEF cell lines. MiRNAs that were differentially expressed were marked by red lines, and *miR*-*10a* was used as a loading control. C: Ten of the 15 miRNAs showed significantly differential expression in both Ptc-/- and Sufu-/- MEF cell lines. They were *let*-*7e, miR*-*30b, miR*-*30c, miR*-*96, miR*-*129*-*3p, miR*-*132, miR*-*134, miR*-*135a, miR*-*143* and *miR*-*183*, respectively. Total RNAs were extracted from the Sufu-/- and WT MEF cells and the expression of 15 miRNAs was detected by the stem-loop RT-PCR, which was done in twice independently. The RT-PCR result was quantified by ImageJ. Error bars represent SD, *P* < 0.017.

Ptc and Sufu are two important suppressors of the Hh signaling pathway. When one of the two genes is knocked out, the Hh signaling pathway will be activated. Gli1, the downstream transcription factor of Hh pathway, can be considered as the marker of the Hh pathway activity[17]–[21]. At first, we detected the Gli1 expression in WT MEF, Ptc-/- MEF and Sufu-/- MEF cells, and found that the Gli1 mRNA levels were higher in the two knockout cell lines; in other words, the Hh pathway was activated in them (***Fig. 1A***). Then, we tested the expression of 90 different miRNAs in WT MEF and Ptc-/- MEF cell lines by the stem-loop RT-PCR. The results showed that 15 miRNAs showed differential expression in the two MEF cell lines (***Fig. 1B***). But only 13 miRNAs had significantly differential expression, they were *let-7e, miR-30b, miR-30c, miR-34a, miR-96, miR-129-3p, miR-132, miR-134, miR-135a, miR-143, miR-146a, mkiR-154* and *miR-183*, respectively (***Fig. 1C***). Afterwards, we performed secondary screening in Sufu-/- MEF cells through the stem-loop RT-PCR. The 15 miRNAs from primary screening were tested and the result was quantified with ImageJ. Of the miRNAs examined, 10 had the same expression trend and significant difference in the two knockout cell lines, and they were *let*-*7e, miR*-*30b, miR*-*30c, miR*-*96, miR*-*129*-*3p, miR*-*132, miR*-*134, miR*-*135a, miR*-*143* and *miR*-*183*, respectively (***Fig. 1C***).

### Quantification of expression of miRNAs for validation of screening results in mouse MB and normal cerebellar tissues

**Fig. 2:**
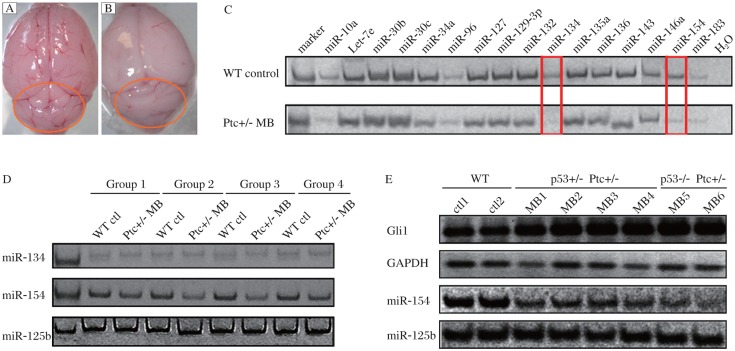
Comparison between normal and medulloblastomas (MB) samples revealed changes in expression of miRNAs. Normal cerebellar tissue (A) and MB (B) are shown here. The areas marked by the red lines indicate the cerebellar tissues, where MB usually develop. C: Fifteen miRNAs identified from the primary screening were tested by stem-loop RT-PCR in the MB and normal cerebellar tissues with *miR*-*10a* as an internal loading control and H_2_O as the negative control. D: Validation of the expression of *miR*-*134* and *miR*-*154* in the other four groups of Ptc+/- MB and WT normal tissues. *MiR*-*125b* was used as a loading control. E: Expression of *miR*-*154* in p53+/- Ptc+/- and p53-/- Ptc+/- MB. Gli1 expression was also detected. *MiR*-*125b* was used as a loading control. WT: wildtype; ctl: control.

It is well known that Ptc is an important suppressor of the Hh signaling pathway. MB is extremely frequent in Ptc knockout mice. Ptc-/- mice died at E9.5, while Ptc+/- mice were viable and susceptible to MB[22]–[23]. In the current study, 20%-30% Ptc heterozygous mice developed brain tumors at the age of about 5 weeks. Then, we separated Ptc+/- MB and normal cerebellar tissues from B6/C57 mice (***Fig. 2A*** and ***Fig. 2B***). Total RNAs were isolated from these tissues and the mRNA levels of Gli1 were detected by RT-PCR. The Gli1 mRNA levels of the Ptc+/- MB were higher than those of the normal cerebellums (data not shown). Therefore, it suggested that Hh signaling was overactivated in the tumor tissues. Then, we validated the expression of the 15 miRNAs selected by the primary and secondary screening. The results showed that *miR*-*134* and *miR*-*154* were down-regulated in the MB, but the other miRNAs showed no significant difference in expression (***Fig. 2C***). In order to verify the above result, we examined the expression of *miR*-*134* and *miR*-*154* in the other four groups of tumor and normal tissues. *MiR*-*154* was down-regulated in the other four MB, but the change of *miR*-*134* was insignificant (***Fig. 2D***).

p53 is a tumor suppressor protein. The frequency of MB in Ptc+/- mice was increased by a *p53* mutant background[24]. Indeed, there were about 70%-80% incidence rate of MB in the *p53* and *Ptc* double knockout mice. In order to further validate the results in the Ptc+/- mice, we repeated the experiment in MB with p53+/- Ptc+/- and p53-/- Ptc+/-. The Hh signaling pathway was activated in *p53* and *Ptc* double knockout MB with a dramtically decrease of *miR*-*154* levels (***Fig. 2E***). This result indicated that the expression of *miR*-*154* was also significantly down-regulated in the MB which were induced by activated Hh pathway activity. MiR-154 may play an important role in the formation of MB induced by activated Hh signaling.

### Expression profile of the *miR*-*154* cluster during the genesis of MB

**Fig. 3:**
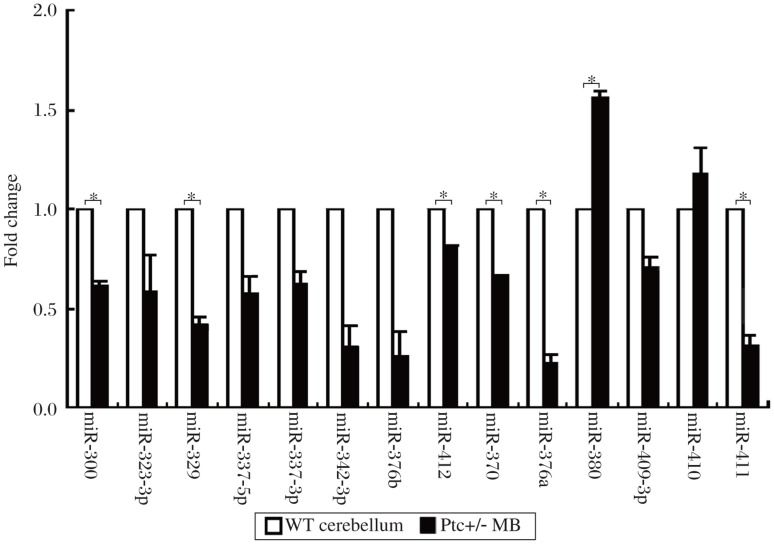
Relative expression of 14 members of the *miR*-*154* cluster in Ptc+/- medulloblastomas (MB) and wildtype (WT) normal cerebellar tissues. The RT-PCR result was quantified by ImageJ software. Six of them were down-regulated, while 1 of them was up-regulated in the MB. This experiment was done twice independently. Error bars represent SD, *P < 0.05.

Mouse genomic structure analysis showed that *miR*-*134* and *miR*-*154* were located in the same miRNA cluster on chromosome 12. The *miR*-*154* cluster is a very huge and complicated gene cluster, including more than 40 miRNA members. To make clear whether other members of the *miR*-*154* cluster had the same expression profile in MB, we detected the other 21 members of the *miR*-*154* cluster by the stem-loop RT-PCR. Seven of them were undetectable because of low expression levels (data not shown). Of the 14 miRNAs, 6 were down-regulated, and 1 was up-regulated in the MB tissues (***Fig. 3***). The result indicated that not only *miR*-*154* but also the whole cluster was regulated by the Hh signaling pathway, and *miR*-*154* may play an important role during the genesis of MB.

## DISCUSSION

MiRNAs play a critical role in developmental and physiologic processes and are involved in the pathogenesis of several human diseases including cancer[25]–[27]. Recently, the relationship between the Hh signaling pathway and miRNAs has been reported[13]–[15]. Search of miRNAs that are regulated by the Hh pathway can help us better understand the Hh pathway, clarify the molecular mechanism of MB, and finally explore new therapeutic targets to cure diseases associated with the Hh pathway.

We selected different MEF cell lines to carry out screening because they are sensitive to the Hh signaling pathway. In the current study, we found that 13 miRNAs showed significantly differential expression between the WT MEF and Ptc-/- MEF cell lines. Then, we performed secondary screening in the Sufu-/- MEF cell lines and the result showed that 10 of the 15 miRNAs had the same expression trend both in Ptc-/- MEF and Sufu-/- MEF cell lines. These 10 miRNAs are interesting because they probably are regulated by the Hh pathway. However, it is still unknown whether they are directly regulated by the Hh pathway or not. This needs more studies on gene transcription regulation. The expressly of the other 5 miRNAs were inconsistent in the Ptc-/- and Sufu-/- MEF cell lines, and it may be falsely positive in the screening.

Subsequently, we verified the 15 miRNA candidates in mouse MB which were induced by activated Hh signaling and found that the expression of *miR*-*154* was significantly lower in the tumor samples than in the normal tissues, in spite of the genotypes. Our study indicated that *miR*-*154* was closely related to the Hh signaling pathway, and it played an essential role in the genesis of MB induced by activated Hh signaling. There are many miRNAs in the *miR*-*154* cluster, but the other members may not be involved in this process.

MB is the most common brain tumor in children, accounting for 10%-20% of the central nervous system tumors. According to the statistics, the peak age of onset of MB is 7 years. Clinically, MB is a highly malignant tumor with aggressive biological behavior and high mortality rate[28]. To improve the survival rate, investigators have carried out numerous studies at the molecular level to explore the pathogenesis of this tumor. Many studies show that the vast majority of MB are induced by sustained activation of the Hh pathway, but the molecular mechanism of MB occurrence has not been characterized yet. We tried to do some research on this problem from the angle of miRNAs.

The *miR*-*154* cluster, located in the human imprinted 14q32 domain (mouse chromosome 12F2), is a very conservative miRNA cluster in mammalians[29]. It is also a very complex and large cluster, including more than 40 members spanning over a distance of over 40 kb. Because of its complex structure, the current study of the *miR*-*154* cluster is relatively limited. The transcription regulation of this cluster is poorly known. It has been considered to play a role in the epigenetic modifications and regulation of embryonic development[30],[31]. In this study, we reported that the *miR*-*154* cluster played a role in medulloblastoma genesis, but it requires further studies on the regulation mechanism of *miR*-*154* by the Hh signaling pathway and further validation of our screening results in more types of tumor.
